# Draft Genome Sequence of *Paenibacillus polymyxa* KF-1, an Excellent Producer of Microbicides

**DOI:** 10.1128/genomeA.00727-16

**Published:** 2016-08-04

**Authors:** Yumei Li, Qiang Li, Yamei Li, Juan Gao, Xiangyu Fan

**Affiliations:** School of Biological Science and Biotechnology, University of Jinan, Jinan, China

## Abstract

We report here the draft genome sequence of *Paenibacillus polymyxa* KF-1, which exhibits excellent antimicrobial activity. It encodes the synthase of bacitracin, kalimantacin, bacillomycin, iturin, fusaricidin, tridecaptin, and pelgipeptin and biosynthetic pathways of antiviral curldan and levan polysaccharides. Also, a novel prophage is involved in this genome that contains endolysin-encoding genes.

## GENOME ANNOUNCEMENT

*Paenibacillus polymyxa* KF-1 was isolated from soil by our lab and was found to be able to produce many kinds of bioactive compounds and show a broad spectrum of antimicrobial activity (our unpublished data). Here, we report the draft genome sequence for further studies focusing on genes or gene clusters mining for the biosynthesis of these functional molecules. 

Genomic DNA of *P. polymyxa* KF-1 was extracted and sequenced using an Illumina MiSeq platform. Shotgun sequencing produced 3,797,994 paired-end reads with approximately 154-fold coverage. Filtered reads were assembled by Newbler 2.8, and gaps were filled by GapCloser (http://soap.genomics.org.cn/about.html). This assembly generated 113 contigs with a total sequence length of 5,784,494 bp, which were assembled into 39 scaffolds with a total sequence length of 5,765,823 bp. The draft genome had an average G+C content of 45% and contained 5,229 open reading frames (ORFs) that were predicted by Glimmer 3.0 (1). The genome annotation was performed via search against the GenBank, UniProt, Pfam, KEGG, COG, and InterPro databases, which revealed rich sources of bioactive compounds. In addition, 107 tRNAs, 6 rRNA loci, and 68 other noncoding RNAs (ncRNAs) were identified via the tRNAsan-SE 1.3.1, RNAmmer 1.2 servers, and Rfam database, respectively.

antiSMASH (http://antismash.secondarymetabolites.org/) analysis showed that the genome contained 17 gene clusters encoding nonribosomal peptide synthases including bacitracin, kalimantacin, bacillomycin, iturin, fusaricidin, tridecaptin, pelgipeptin, and an additional two polyketide synthases. An NCBI conserved domains search revealed β-1,3-glycanases (curldan synthases) and levansucrases for biosynthesis of antiviral curldan and leven polysaccharides (2, 3). PHAge Search Tool (http://phast.wishartlab.com/index.html) scanning indicated that there was a 5.9-kb full-length prophage genome.

### Nucleotide sequence accession numbers.

This whole-genome shotgun project has been deposited at DDBJ/EMBL/GenBank under the accession no. LNZF00000000. The version described in this paper is the first version, LNZF00000000.1. The BioProject number is PRJNA304353. The Ref-Seq accession number is NZ_ LNZF01000000.

## References

[1] Delcher AL, Harmon D, Kasif S, White O, Salzberg SL 1999 Improved microbial gene identification with Glimmer. Nucleic Acids Res 27:4636–4641. doi:10.1093/nar/27.23.4636. 10556321 PMC148753

[2] Esawy MA, Abdel-Fattah AM, Ali MM, Helmy WA, Salama BM, Taie HA, Hashem AM, Awad GE 2013 Levansucrase optimization using solid state fermentation and levan biological activities studies. Carbohydr Polym 96:332–341. doi:10.1016/j.carbpol.2013.03.089. 23688489

[3] Periasamy A, Shadiac N, Amalraj A, Garajová S, Nagarajan Y, Waters S, Mertens HD, Hrmova M 2013 Cell-free protein synthesis of membrane (1,3)-β-d-glucan (curdlan) synthase: co-translational insertion in liposomes and reconstitution in nanodiscs. Biochim Biophys Acta 1828:743–757. doi:10.1016/j.bbamem.2012.10.003. 23063656

